# Enhancement of solubility in *Escherichia coli *and purification of an aminotransferase from *Sphingopyxis *sp. MTA144 for deamination of hydrolyzed fumonisin B_1_

**DOI:** 10.1186/1475-2859-9-62

**Published:** 2010-08-18

**Authors:** Doris Hartinger, Stefan Heinl, Heidi Elisabeth Schwartz, Reingard Grabherr, Gerd Schatzmayr, Dietmar Haltrich, Wulf-Dieter Moll

**Affiliations:** 1 BIOMIN Research Center, Technopark 1, 3430 Tulln, Austria; 2 Institute of Applied Microbiology, Department of Biotechnology, University of Natural Resources and Life Sciences, Muthgasse 18, 1190 Vienna, Austria; 3 Center for Analytical Chemistry, Department for Agrobiotechnology IFA-Tulln, University of Natural Resources and Life Sciences, Vienna, Konrad Lorenz Strasse 20, 3430 Tulln, Austria; 4 Food Biotechnology Laboratory, Department of Food Sciences and Technology, University of Natural Resources and Life Sciences, Muthgasse 18, 1190 Vienna, Austria

## Abstract

**Background:**

Fumonisin B_1 _is a cancerogenic mycotoxin produced by *Fusarium verticillioides *and other fungi. *Sphingopyxis *sp. MTA144 can degrade fumonisin B_1_, and a key enzyme in the catabolic pathway is an aminotransferase which removes the C2-amino group from hydrolyzed fumonisin B_1_. In order to study this aminotransferase with respect to a possible future application in enzymatic fumonisin detoxification, we attempted expression of the corresponding *fumI *gene in *E. coli *and purification of the enzyme. Since the aminotransferase initially accumulated in inclusion bodies, we compared the effects of induction level, host strain, expression temperature, solubility enhancers and a fusion partner on enzyme solubility and activity.

**Results:**

When expressed from a T7 promoter at 30°C, the aminotransferase accumulated invariably in inclusion bodies in DE3 lysogens of the *E. coli *strains BL21, HMS174, Rosetta 2, Origami 2, or Rosetta-gami. Omission of the isopropyl-beta-D-thiogalactopyranoside (IPTG) used for induction caused a reduction of expression level, but no enhancement of solubility. Likewise, protein production but not solubility correlated with the IPTG concentration in *E. coli *Tuner(DE3). Addition of the solubility enhancers betaine and sorbitol or the co-enzyme pyridoxal phosphate showed no effect. Maltose-binding protein, used as an N-terminal fusion partner, promoted solubility at 30°C or less, but not at 37°C. Low enzyme activity and subsequent aggregation in the course of purification and cleavage indicated that the soluble fusion protein contained incorrectly folded aminotransferase. Expression in *E. coli *ArcticExpress(DE3), which co-expresses two cold-adapted chaperonins, at 11°C finally resulted in production of appreciable amounts of active enzyme. Since His tag-mediated affinity purification from this strain was hindered by co-elution of chaperonin, two steps of chromatography with optimized imidazole concentration in the binding buffer were performed to obtain 1.45 mg of apparently homogeneous aminotransferase per liter of expression culture.

**Conclusions:**

We found that only reduction of temperature, but not reduction of expression level or fusion to maltose-binding protein helped to produce correctly folded, active aminotransferase FumI in *E. coli*. Our results may provide a starting point for soluble expression of related aminotransferases or other aggregation-prone proteins in *E. coli*.

## Background

**Figure 1:**
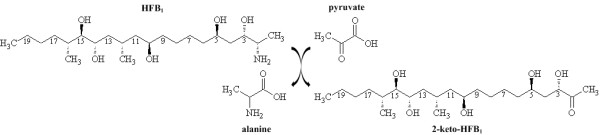
**The reaction catalyzed by aminotransferase FumI**. The enzyme transfers the 2-amino group from hydrolyzed fumonisin B_1 _(HFB_1_, 2-Amino-12,16-dimethylicosane-3,5,10,14,15-pentol) to pyruvate, producing 2-keto-HFB_1 _(3,5,10,14,15-Pentahydroxy-12,16-dimethylicosane-2-one) and alanine.

The aminotransferase FumI from the bacterium *Sphingopyxis *sp. MTA144 [1] may be useful in a technological application for detoxification of the mycotoxin fumonisin B_1 _(FB_1_). FB_1 _is the most prevalent member of the fumonisin mycotoxin family [2], which is predominantly produced by *Fusarium verticillioides *(former *F. moniliforme*) and *F. proliferatum*, and frequently found in maize and maize products from warm climate regions of the world [3,4]. Since their isolation and structure determination in 1988 [5,6], the toxic and carcinogenic effects of fumonisins, in particular FB_1_, have been demonstrated in several studies [7-17]. Approaches to reduce fumonisin contamination in food and feed included various chemical and physical strategies [18-24]. However, none of these methods have been implemented on a large scale, and fumonisins in the diet still affect the health of livestock [25] and possibly also of humans. Our technological goal is to provide fumonisin-degrading enzymes as a feed additive for detoxification in the gastrointestinal tract of animals. Genes and enzymes for fumonisin detoxification from yeast strains [26], an unidentified bacterial strain [27], and *Sphingopyxis *sp. MTA144 [1] have previously been described. The catabolic pathways share the initial hydrolytic cleavage of the two tricarballylic acid side chains from C14 and C15 of the main chain of fumonisin B_1_. The reaction product, hydrolyzed FB_1 _(HFB_1_), has reduced affinity to the primary target of FB_1_, the enzyme ceramide synthase [28,29]. However, the amino group of HFB_1 _can be acylated by ceramide synthase, forming toxic metabolites [30,31]. In contrast to the yeast strains, which use an amino oxidase, *Sphingopyxis *sp. MTA144 uses the aminotransferase FumI for deamination of HFB_1_, resulting in the formation of 2-keto-HFB_1 _(Fig. 1). Since this aminotransferase, unlike the amino oxidase, does not require molecular oxygen, it may be suitable for HFB_1 _detoxification in the gastrointestinal tract. Therefore we intended to prepare pure and active recombinant aminotransferase FumI for studying its technological potential as a feed enzyme. Initially, we found that expression of a codon-optimized version of the *fumI *gene in *Pichia pastoris *gave very low enzyme yields, and efforts to obtain active enzyme by refolding from inclusion bodies isolated from *E. coli *remained unsuccessful. Even though the enzyme aggregated in *E. coli*, the aminotransferase FumI activity was detectable in clarified cell lysate, which motivated our strategy of trying to enhance solubility. Several strategies have been described to obtain soluble expression in *E. coli *[32], including the use of solubility-enhancing fusion tags [33,34], expression at reduced temperature [35], co-expression of chaperones [36], reduction and fine-tuning of expression level [37], export to the periplasm [38,39] or change of the cytoplasmic redox potential for disulfide bond formation [40], consideration of codon usage [41], optimization of bioprocess parameters, or use of dedicated screening tools for finding conditions for soluble expression [42]. Since factors leading to recombinant protein aggregation are not only host-specific but also target protein specific, no universal remedy for inclusion body formation is available. For the present report, we explored several approaches of increasing the yield of soluble and active aminotransferase FumI in *E. coli*, including comparison of several *E. coli*(DE3) strains, isopropyl-beta-D-thiogalactopyranoside (IPTG) concentrations and expression temperatures, and fusion with maltose-binding protein (MBP). The best yield, sufficient for His tag-mediated purification using an optimized protocol, was obtained by expression in *E. coli *ArcticExpress(DE3) at low temperature.

## Results

### Comparison of *E. coli*(DE3) strains

**Figure 2:**
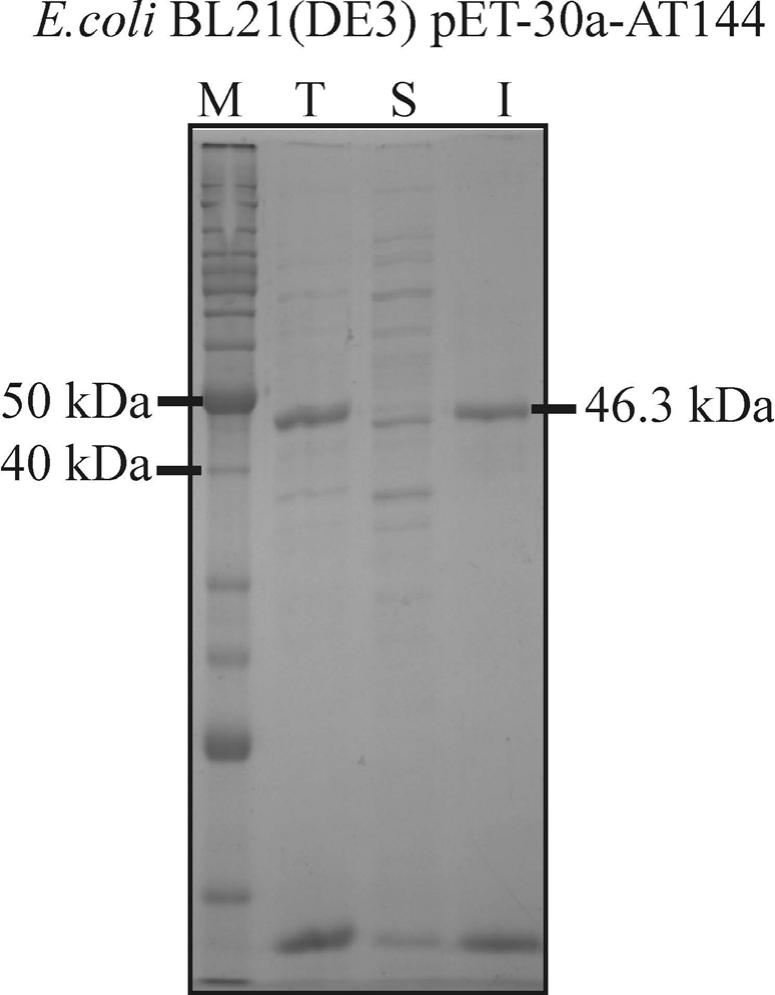
**Expression of *fumI *in *E. coli *BL21(DE3) at 37°C**. SDS-PAGE (12% polyacrylamide, GelCode Blue staining) analysis of fractions of cell lysate of *E. coli *BL21(DE3) expressing *fumI *from vector pET-30a-AT144 by induction with 1 mM IPTG at 37°C. Lane M, molecular mass marker; Lane T, total cell lysate; Lane S, soluble fraction of cell lysate; Lane I, insoluble fraction. The molecular mass of aminotransferase FumI (46.3 kDa) is indicated.

**Figure 3:**
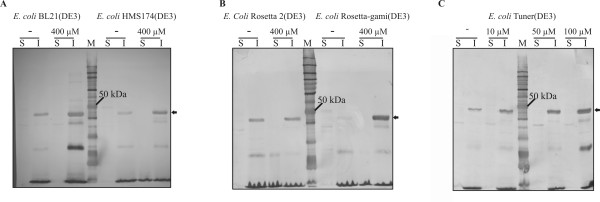
**Comparison of *E. coli*(DE3) host strains and effect of IPTG concentrations on FumI production at 30°C**. Western blot (anti-His_6_-antibody; from 12% polyacrylamide SDS-PAGE gels) analysis of N-terminally 6xHis-tagged aminotransferase FumI. The *fumI *gene was expressed in *E. coli *BL21(DE3) (A), *E. coli *HMS174(DE3) (A), *E. coli *Rosetta 2(DE3) (B), *E. coli *Rosetta-gami(DE3) (B) and *E. coli *Tuner(DE3) (C) from vector pET-3a-HISAT at 30°C without (-) and with addition of IPTG (10 μM, 50 μM, 100 μM, 400 μM). Lane S, soluble fraction of cell lysate; Lane I, insoluble fraction; Lane M, molecular mass marker. The arrow indicates the band corresponding to the molecular mass of His-tagged aminotransferase FumI (47.5 kDa).

The combinations of different expression vectors and *E. coli *strains used in this work are listed in Table 1. Since the *fumI *gene contains a restriction site for *Nco*I, pET vectors that enable gene insertion at an *Nde*I site covering the translation initiation codon were chosen. Initially, the *fumI *gene was cloned into vector pET-30a(+) for low background expression based on the T7*lac *promoter. When the gene was expressed in *E. coli *BL21(DE3) by induction with 1 mM IPTG at 37°C, most of the aminotransferase was found in the insoluble fraction of cell lysate (Fig. 2). Since our efforts to refold the aminotransferase from solubilized inclusion bodies by dialysis or dilution remained unsuccessful, we attempted to increase the yield of soluble, correctly folded protein produced in recombinant *E. coli*. We had previously observed when using other recombinant proteins that *E. coli*(DE3) strains can produce considerable amounts of recombinant protein from a gene cloned under control of a T7 promoter even in the absence of IPTG. So the *fumI *gene was cloned into vector pET-3a, which does not contain a *lac *operator sequence downstream of the T7 promoter. *E. coli *BL21(DE3) harboring plasmid pET-3a-AT or pET-3a-HISAT was grown at 37°C without IPTG for low level recombinant gene expression, which might be beneficial for correct folding. However, aminotransferase FumI still accumulated in inclusion bodies. Addition of 2.5 mM betaine or 600 mM sorbitol, which were previously proposed as solubility enhancers [43], to LB medium for expression failed to increase the yield of soluble aminotransferase. Pyridoxal phosphate, which is a co-enzyme of aminotransferase FumI, likewise showed no effect when added to the medium at a concentration of 1 mM. Subsequently, we reduced the expression temperature to 30°C, and compared the *E. coli *strains BL21(DE3), HMS174(DE3), Tuner(DE3), Rosetta-gami(DE3), Origami 2(DE3) and Rosetta 2(DE3) with respect to expression of active FumI. However, none of these experiments resulted in the expression of significant amounts of soluble FumI, both when 0.4 mM IPTG or no induction with IPTG were used, and the recombinant His-tagged protein was only seen in the insoluble fraction (Fig. 3A, B). When using *E. coli *Tuner(DE3), which is a *lacZY *deletion mutant that allows a concentration-dependent level of induction with IPTG since the permease LacY is lacking, a correlation between IPTG concentration and expression level was observed. Yet again, reduction of the expression level failed to enhance the yield of soluble aminotransferase (Fig. 3C).

**Table 1:** Recombinant *E. coli *strains used in this work. AT or AT144 was used for *fumI *in the vector names.

Host strain	Recombinant Plasmid	Fusion tag	Recombinant strain
***E. coli *BL21(DE3)**	pET-3a-AT	**-**	*E. coli *BL21(DE3) pET-3a-AT
	pET-3a-HISAT	N-terminal 6xHis tag	*E. coli *BL21(DE3) pET-3a-HISAT
	pET-30a-AT144	-	*E. coli *BL21(DE3) pET-30a-AT144
	pMAL-AT144	MBP	*E. coli *BL21(DE3) pMAL-AT144

***E. coli *HMS174(DE3)**	pET-3a-HISAT	N-terminal 6xHis tag	*E. coli *HMS174(DE3) pET-3a-HISAT
	pMAL-AT144	MBP	*E. coli *HMS174(DE3) pMAL-AT144

***E. coli *Origami 2(DE3)**	pET-3a-HISAT	N-terminal 6xHis tag	*E. coli *Origami 2(DE3) pET-3a-HISAT
	pMAL-AT144	MBP	*E. coli *Origami 2(DE3) pMAL-AT144
	pMAL		*E. coli *Origami 2(DE3) pMAL

***E. coli *Rosetta 2(DE3)**	pET-3a-HISAT	N-terminal 6xHis tag	*E. coli *Rosetta 2(DE3) pET-3a-HISAT

***E. coli *Rosetta-gami(DE3)**	pET-3a-HISAT	N-terminal 6xHis tag	*E. coli *Rosetta-gami(DE3) pET-3a-HISAT

***E. coli *Tuner(DE3)**	pET-3a-HISAT	N-terminal 6xHis tag	*E. coli *Tuner(DE3) pET-3a-HISAT

***E. coli *ArcticExpress(DE3)**	pET-30a-AT144HIS	C-terminal 6xHis tag	*E. coli *ArcticExpress(DE3) pET-30a-AT144HIS
	pET-3a-HISAT	N-terminal 6xHis tag	*E. coli *ArcticExpress(DE3) pET-3a-HISAT
	pET-3a-AT	-	*E. coli *ArcticExpress(DE3) pET-3a-AT

### Expression and purification of aminotransferase FumI as fusion protein with maltose-binding protein

**Figure 4:**
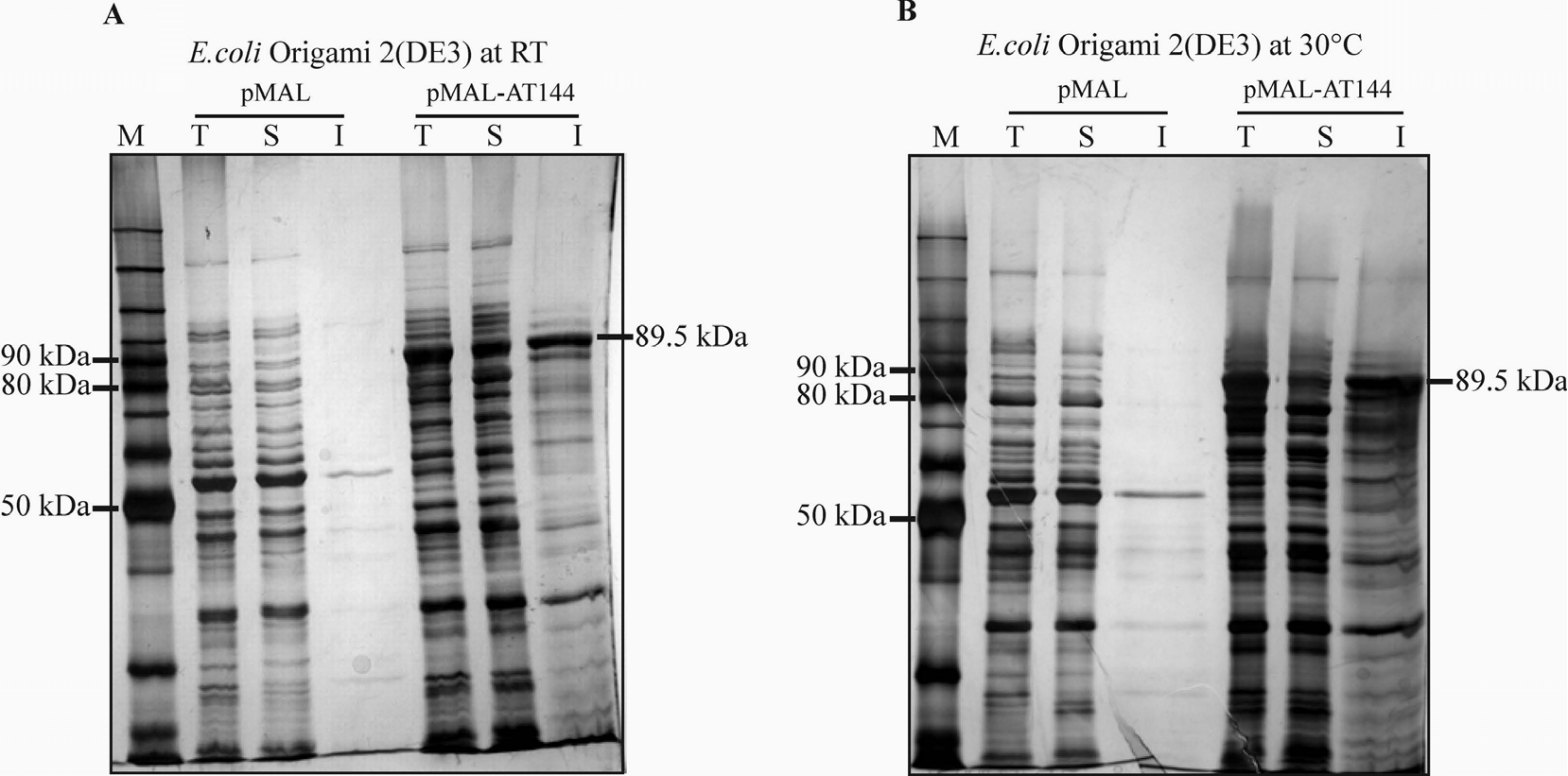
**Solubility of MBP-FumI fusion protein produced in *E. coli*(DE3) at 30°C or 23.5°C**. SDS-PAGE (10% polyacrylamide, silver staining) analysis of total (T), soluble (S) and insoluble (I) fractions of cell lysates of *E. coli *Origami 2(DE3) after expression of MBP-FumI fusion protein (89.5 kDa) from vector pMAL-AT144, or MBP (50.9 kDa) from vector pMAL (pMAL-c2E, no insert). Expression was done for 20 h at room temperature (RT, 23.5°C) (A) or 30°C (B). Lane M, molecular mass marker.

**Figure 5:**
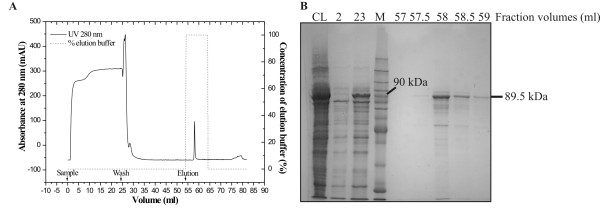
**Purification of MBP-FumI fusion protein by affinity chromatography**. Chromatogram (A) of affinity chromatography of MBP-FumI fusion protein (89.5 kDa) on dextrin sepharose and elution with 50 mM maltose, and analysis of fractions by SDS-PAGE (10% polyacrylamide, GelCode Blue staining) (B). Lane CL, clarified cell lysate; lane 2, fraction from 2 to 2.5 ml; lane 23, fraction from 23 to 23.5 ml and so forth; lane M, molecular mass marker.

**Figure 6:**
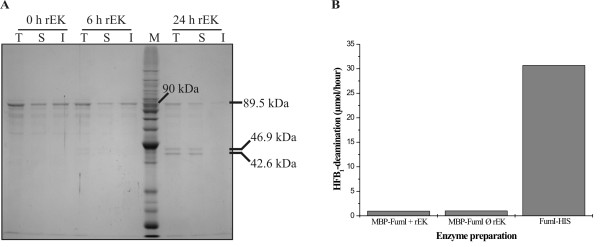
**Enterokinase cleavage and aminotransferase activity of purified MBP-FumI fusion protein**. (A) Total (T), soluble (S) and insoluble (I) fractions of purified MBP-FumI fusion protein before addition of recombinant enterokinase (0 h rEK), after 6 h and after 24 h incubation with recombinant enterokinase were subjected to SDS-PAGE (10% polyacrylamide, GelCode Blue staining). Lane M, molecular mass marker. The molecular masses of MBP (42.6 kDa), FumI (46.9 kDa) and the fusion protein (89.5 kDa) are indicated. (B) Enzyme activity of purified MBP-FumI fusion protein after incubation with (+ rEK) or without (Ø rEK) recombinant enterokinase. For comparison, 6xHis-tagged aminotransferase (FumI-HIS) purified from *E. coli *ArcticExpress(DE3) by affinity chromatography was included. Protein concentration was 20 μg/ml for each sample. Samples were incubated with 15 μM HFB_1 _in 20 mM Tris-HCl buffer (pH 8.0) at 25°C, and initial enzyme velocities, based on HFB_1 _deamination, were plotted.

Several examples have been reported, in which the use of MBP as a fusion partner effectively promoted the expression of soluble recombinant protein in *E. coli *[44-47]. In order to test this possibility for FumI, we constructed plasmid pMAL-AT144 for production of the MBP-FumI fusion protein. When the expression temperature was 37°C, the fusion protein accumulated in the insoluble fraction of cell lysates of *E. coli *BL21(DE3), HMS174(DE3) and Origami 2(DE3). However, when the expression temperature was reduced to 30°C or room temperature, and the expression time was concomitantly extended from 2 to 20 h, *E. coli *Origami 2(DE3) produced some of the fusion protein in soluble form (Fig. 4). As judged from the intensities of the protein bands, expression at room temperature yielded more soluble fusion protein than at 30°C. The fusion protein was subsequently purified by affinity chromatography on dextrin sepharose resin (Fig. 5), and approximately 0.8 mg of purified MBP-FumI fusion protein could be obtained per liter of expression culture. When the sample was analyzed after dialysis against the buffer required for recombinant enterokinase cleavage, part of the fusion protein was found to be aggregated. However, after cleavage with recombinant enterokinase, both fusion partners were found in the soluble fraction (Fig. 6A). The enzymatic activity of the fusion protein was lower than that of the purified His-tagged enzyme and did not increase upon separation of the fusion partners with enterokinase (Fig. 6B).

### Expression in *E. coli *ArcticExpress(DE3) and His tag-mediated affinity purification

**Figure 7:**
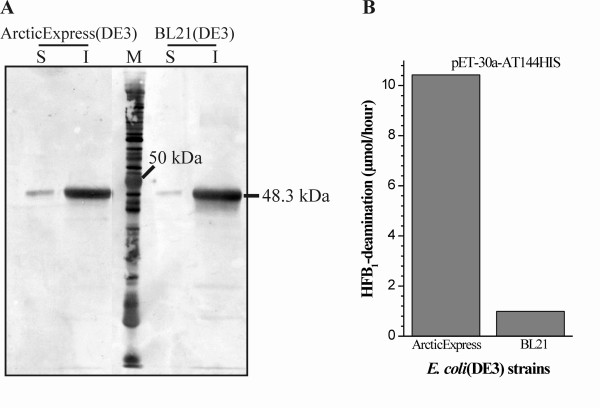
**Comparison of solubility and activity of FumI produced in *E. coli *strains ArcticExpress(DE3) at 11°C and BL21(DE3) at 37°C**. Aminotransferase FumI showed a higher amount of soluble enzyme in a Western blot (anti-His_6_-antibody; from 12% polyacrylamide SDS-PAGE gel) (A) and higher enzyme activity (B) when produced in *E. coli *ArcticExpress(DE3) at 11°C compared with *E. coli *BL21(DE3) at 37°C. Both host strains harbored plasmid pET-30a-AT144HIS for expression of *fumI *with a C-terminal 6xHis tag. (A) The molecular mass of aminotransferase FumI-HIS (48.3 kDa) is shown; (S) soluble and (I) insoluble fractions of *E. coli *cell lysates. (B) Initial enzyme velocities based on HFB_1 _deamination.

**Figure 8:**
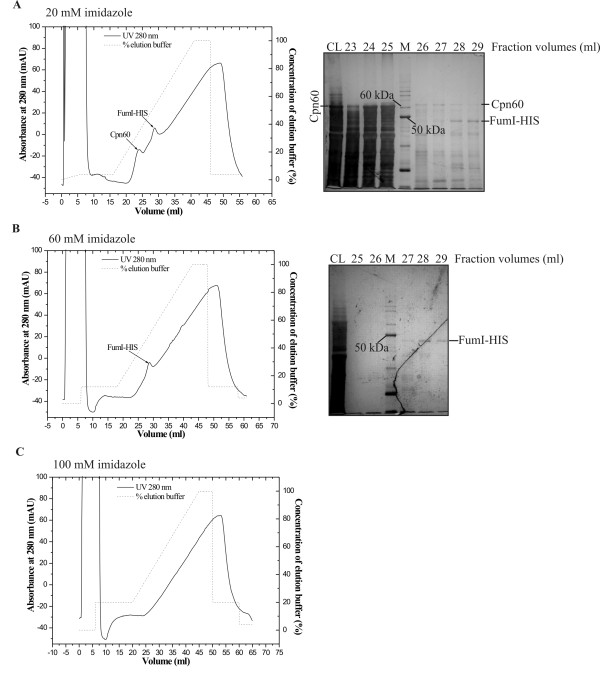
**Purification of C-terminally 6xHis-tagged aminotransferase FumI by nickel affinity chromatography**. Cell extract of *E. coli *ArcticExpress(DE3) [pET-30a-AT144HIS] after production of FumI-HIS (48.3 kDa) was subjected to nickel affinity chromatography using 20 mM (A), 60 mM (B) or 100 mM (C) imidazole in binding buffer. SDS-PAGE analysis (12% polyacrylamide, Coomassie Brilliant Blue stained) of samples corresponding to these chromatograms show the clarified cell lysate that was applied to the column (CL), samples of the 1-ml fractions that eluted after the indicated volume, and a molecular mass marker (M).

As described above, the fraction of soluble recombinant protein increased when the expression temperature was reduced. Therefore, we attempted a further reduction of temperature by expressing *fumI *in *E. coli *ArcticExpress(DE3). This strain heterologously produces chaperonin Cpn60 and co-chaperonin Cpn10 from the psychrophilic bacterium *Oleispira antarctica*, which both show high protein refolding activities at 4°C to 12°C and allow *E. coli *to grow at high rates (0.28 to 0.45 h^-1^) at 4°C to 15°C [48]. Vector pET-30a-AT144HIS was used for expression of *fumI *in *E. coli *ArcticExpress(DE3) at 11°C for 24 h. Most of the gene product still aggregated to insoluble inclusion bodies, however, as judged from the intensities of protein bands and enzyme activity measurements, more soluble protein was obtained when compared to expression from the same vector in *E. coli *BL21(DE3) at 37°C for 3 h (Fig. 7). When we purified recombinant aminotransferase from clarified cell lysates by immobilized nickel affinity chromatography, foreign protein co-eluted with the His-tagged enzyme. This protein was likely to be Cpn60 because of its molecular mass, and because chaperonins are known to be co-purified with various proteins when using immobilized metal affinity chromatography (IMAC) [49-51]. In order to obtain homogenous enzyme preparations, variation of the imidazole concentration in the binding buffer was studied. Co-purification of Cpn60 could be circumvented when using 60 mM imidazole in the binding buffer, while 100 mM imidazole prevented binding of both Cpn60 and His-tagged aminotransferase to the chromatography matrix (Fig. 8).

**Figure 9:**
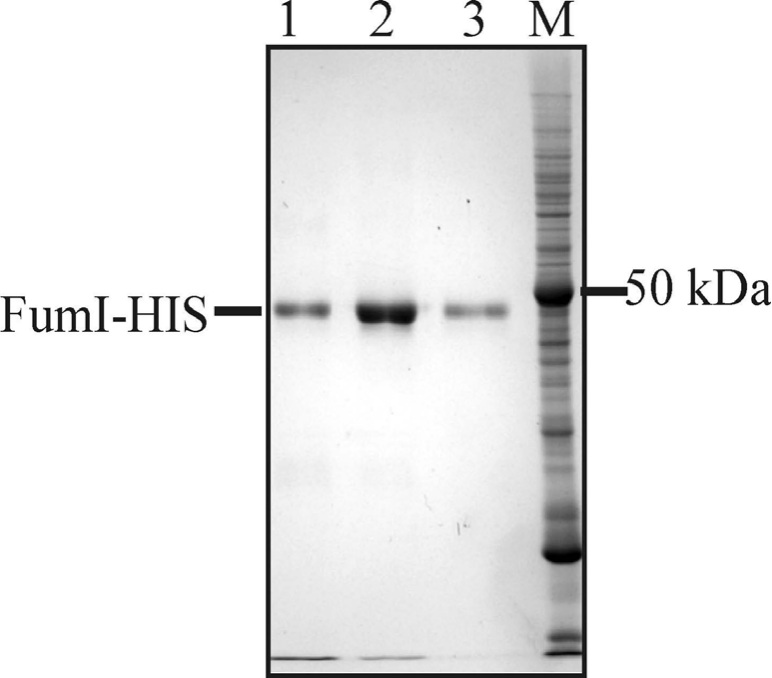
**SDS-PAGE analysis of purified aminotransferase FumI**. The three lanes show three neighboring fractions of C-terminally 6xHis-tagged aminotransferase FumI (FumI-HIS, 48.3 kDa) after two consecutive, preparative scale immobilized nickel affinity chromatography steps using 60-mM imidazole binding buffer. SDS-PAGE gel (12% polyacrylamide, Coomassie Brilliant Blue stained); M, molecular mass marker.

For the preparation of larger amounts of FumI, *E. coli *ArcticExpress(DE3) harboring pET-30a-AT144HIS was grown in 1 l of modified M9ZB medium, expression was done for 24 h at 11°C, and a concentration of 60 mM imidazole in the binding buffer was used for subsequent IMAC. Two IMAC steps using conditions as described above gave an enzyme preparation that was apparently homogenous as analyzed by SDS-PAGE (Fig. 9). When using these conditions both for expression and purification, approximately 1.45 mg of purified aminotransferase FumI could be obtained per liter of expression culture.

## Discussion

The use of feed enzymes for gastrointestinal detoxification of fumonisins may be a practical solution for ameliorating the negative health effects in animal nutrition caused by fumonisin-contaminated maize, which is frequently encountered in the warmer regions of the world [3]. Detoxification of mycotoxins by biodegradation with specific microorganisms has previously been proposed [52-54]. However, application of one of the known yeast [26] or bacterial [1,27,55] strains capable of fumonisin catabolism as a feed additive would require thorough investigation of possible feed safety issues, and pose the technological challenge of providing viable, yet shippable and storable microbes with sufficient activity for fast fumonisin degradation in the gastrointestinal tract of domestic animals. In comparison, enzymatic detoxification would be a safe and effective alternative, if the specific enzymes can be produced with high yield in microbial cell factories, and if they are active in the gastrointestinal tract. Several enzymes, including phytase [56], are commercially provided as feed additive. In order to evaluate whether the aminotransferase FumI encoded by the *fumI *gene of *Sphingopyxis *sp. MTA144 [1] would be active under the conditions in the gastrointestinal tract, or in steps of feed and possibly also food processing, it was essential to have sufficient amounts of purified enzyme preparations for subsequent studies available. The transition from the unfolded polypeptide to the correctly folded native state may be difficult for FumI, since we consistently failed to produce active enzyme by refolding of inclusion bodies using several protocols and including co-enzyme, substrate and co-substrate. This ineptness in folding could also be a reason why expression of a codon-optimized version of the *fumI *gene in *Pichia pastoris *yielded barely detectable amounts of the active aminotransferase. Even though virtually all of the recombinant FumI protein was found in the sedimenting fraction of cell lysate in the initial efforts to produce the aminotransferase in *E. coli*, low aminotransferase activity was detectable in clarified cell lysate. This indicated that in principle correctly folded aminotransferase could be produced in *E. coli*, and this was the basis for further exploring *E. coli *as host for soluble expression of active aminotransferase.

Since expression using the vectors pET-3a-AT or pET-3a-HISAT in the absence of IPTG in *E. coli *BL21(DE3) or with varying IPTG concentrations in *E. coli *Tuner(DE3) showed no noticeable effect on the amount or relative proportion of soluble aminotransferase synthesized, we concluded that solubility of aminotransferase FumI does not correlate with expression levels. Promoter strength and expression level are considered important factors for the cytoplasmic solubility of recombinant proteins [37], and a correlation between induction level and solubility of recombinant proteins in the *E. coli *cytoplasm has been reported [57]. Our results are more in line with previous findings that the percent solubility was independent of recombinant protein concentration or production rate [35]. Inclusion body formation did not seem to be caused by the presence and possible interference with translation of rare codons in the *fumI *gene, since expression in *E. coli *Rosetta 2(DE3), which supplies tRNAs for the rarely used codons AUA, AGG, AGA, CUA, CCC and GGA, did not improve solubility. Efficient folding of recombinant proteins with multiple disulfide bonds in the cytoplasm of *E. coli *mutants *trxB*^- ^(thioredoxin reductase) and *gor*^- ^(glutathion reductase) has been reported [40]. Aminotransferase FumI has eight cysteine residues, but as a cytoplasmic enzyme in *Sphingopyxis *sp. MTA144 it is not predicted by DISULFIND (disulfind.dsi.unifi.it) [58] to have disulfide bonds. Furthermore, the protein with the highest sequence identity (glutamate 1-semialdehyde aminotransferase, 26% identity) in the PDB database does not contain S-S bridges. Failure to enhance solubility by expression of *fumI *in *trxB*^- ^*gor*^- ^*E. coli *Origami 2(DE3) is in agreement with these considerations. The conclusion that *fumI *is a difficult target for soluble and active expression in *E. coli *was further confirmed by the finding that addition of sorbitol or betaine to the expression cultures as osmotic modulators had no effect on aminotransferase activity or solubility. In this respect, aminotransferase FumI behaved differently to several recombinant proteins that were reported to show higher solubility in *E. coli *in the presence of these osmolytes [43,59,60].

Together with full-length aminotransferase, shorter fragments of the enzyme seemed to accumulate upon *fumI *gene expression in *E. coli*. When FumI was produced with an N-terminal His tag, a peptide of less than 15 kDa, which migrated with the dye front or slightly slower and which was detectable in the soluble fraction of some and in the insoluble fraction of all lysates, reacted specifically with the anti-His_6_-antibody. Truncated versions of MBP-FumI were also detectable. Since no such peptides interacted with anti-His_6_-antibody or HisTrap chromatography resin when FumI was produced with a C-terminal His tag, truncated versions may have been formed by premature translation termination.

Fusion of aminotransferase to maltose-binding protein, which was described as more effective in promoting solubility of recombinant proteins than other fusion partners [45], helped to prevent aggregation only at reduced expression temperature but not at 37°C, adding further evidence for the aggregation-prone nature of aminotransferase FumI. It has been reported that maltose-binding protein can keep a fusion partner in solution, even when this partner has not adopted the correct fold [61,62]. There is evidence that this might in part also be the case with MBP-FumI. The soluble fusion protein showed 30-fold lower aminotransferase activity than a preparation of 6xHis-tagged aminotransferase purified from cell extract of *E. coli *ArcticExpress(DE3) when compared at identical concentrations of recombinant protein. Since the enzymatic activity remained unchanged after the fusion partners were separated with recombinant enterokinase, it is possible that the maltose-binding protein fusion does not interfere with aminotransferase activity and that the reduced activity results from incorrectly folded FumI. Further evidence for this is that some of the fusion protein aggregated after affinity purification, when the NaCl concentration was reduced by dialysis and CaCl_2 _was added for subsequent enterokinase cleavage. Under these conditions, neither MBP nor correctly folded aminotransferase aggregate. However, enterokinase cleavage transferred both fusion partners (MBP and FumI) from the sedimentable fraction to the soluble fraction, which does not fit the assumption that incorrectly folded aminotransferase caused aggregation of the fusion protein. A possible explanation could be that the interaction of enterokinase with the polypeptide chain helped to separate, but not refold, aggregates. In experiments that are not shown we observed that after enterokinase cleavage the amount of soluble aminotransferase did not match the amount of MBP in solution, especially when longer incubation times or higher protein concentrations were used. Taken together, fusion with maltose-binding protein seems to have enhanced solubility of aminotransferase FumI more than it promoted correct folding.

Expression temperature has long been known as a key factor determining solubility of recombinant proteins in the cytoplasm of *E. coli *[35,63], and there are numerous examples where growth at low temperature increased solubility. In the case of aminotransferase, a clear effect of temperature reduction on the levels of soluble recombinant protein obtained was only observed when aminotransferase was fused to maltose-binding protein, or produced in *E. coli *ArcticExpress(DE3). The tenfold increase in enzyme activity that we observed when comparing expression from the same plasmid in *E. coli *BL21(DE3) at 37°C and *E. coli *ArcticExpress(DE3) at 11°C was considerably less than e.g. the more than 150-fold increase reported by Ferrer et al. (2004) for the expression of an esterase under similar conditions [64]. Nevertheless, reduction of temperature was the single most effective measure among the approaches we tested to enhance solubility and activity of aminotransferase FumI. Our observation that the co-expressed chaperonin Cpn60 interfered with affinity purification by IMAC and that its elution trailed to the fractions of the recombinant target protein has been described before and was circumvented by changing the binding buffer composition to reduce the interaction between Cpn60 and the recombinant protein [49]. We found that co-elution of Cpn60 could also be avoided by increasing the imidazole concentration in the binding buffer, although at preparative scale a repeat of the affinity chromatography step was necessary to obtain pure target enzyme.

## Conclusions

Correct folding of recombinant aminotransferase FumI in *E. coli *could not be enhanced by reducing the expression level or by fusion to maltose-binding protein. Only lowering the expression temperature to 11°C, enabled by using *E. coli *ArcticExpress(DE3) as a host, helped the formation of active enzyme. The recombinant aminotransferase FumI thus produced and purified was sufficient to obtain apparently homogeneous protein for subsequent determination of enzyme characteristics, kinetic parameters and evaluation of the application potential as feed enzyme (manuscript in preparation). Further reduction of expression temperature may increase the yield of soluble, active enzyme. However, if aminotransferase FumI were to be produced on a technological scale for application as feed enzyme, an alternative and improved expression system will likely be required.

## Methods

### Strains, vectors, chemicals, media

*E. coli *host strains for gene expression BL21(DE3), HMS174(DE3), Rosetta 2(DE3), Origami 2(DE3), Rosetta-gami(DE3) and Tuner(DE3) were purchased from Novagen (Madison, WI, USA), and strain *E. coli *ArcticExpress(DE3) was from Stratagene (La Jolla, CA, USA). The cloning host strain *E. coli *XL1-Blue was from Stratagene. Genetic transformations were performed according to manufacturer's instructions. *Sphingopyxis *sp. MTA144 was previously isolated from a soil sample. The sequence of the *fum *gene cluster was deposited under GenBank accession no. FJ426269[1].

Vector pMAL-c2E was purchased from New England Biolabs (Ipswich, MA, USA), and vectors pET-3a and pET-30a(+) were obtained from Novagen. All chemicals were from Sigma (St. Louis, MO, USA). Enzymes were purchased from Fermentas (St. Leon-Rot, Germany), except for recombinant enterokinase, which was obtained from Novagen.

### Construction of the expression vectors

Molecular cloning was performed according to standard procedures [65], and all plasmids were confirmed by sequencing of the inserts (VBC-Biotech, Vienna, Austria). The expression vector and host strain combinations used in this work are summarized in Table 1.

#### pET-3a-AT and pET-3a-HISAT

Cloning of the aminotransferase gene *fumI *in pET-3a for expression with or without an N-terminal 6xHis tag has previously been described [1].

#### pET-30a-AT144HIS and pET-30a-AT144

The *fumI *gene was amplified by PCR from a preparation of genomic DNA of *Sphingopyxis *sp. MTA144 [1] using the primers NdeI-fumI-for (5'-CTT ATA **CAT *ATG ***GCG AAC GGA ACA AGG CAG AAA G-3') and SacI-fumI-rev (5'-TAT **GAG CTC **GCA GCA CCG GCG AGT TGC ATT AAA ATG G-3'). Restriction sites are in boldface and start or stop codons are in italics. Vector pET-30a(+) and the PCR product were digested with *Nde*I and *Sac*I, and ligated to form pET-30a-AT144HIS. The encoded protein (48.3 kDa) comprises the FumI sequence [GenBank: ACS27061] with a C-terminal extension of ASSVDKLAAALEHHHHHH.

For expression without a 6xHis tag, *fumI *was amplified by PCR with NdeI-fumI-for and SacI-Stop-fumI-rev (5'-TAT **GAG CTC ***TTA *AGC ACC GGC GAG TTG CAT TAA AAT GG-3') and cloned as above to give pET-30a-AT144.

#### pMAL-AT144

The *fumI *gene was amplified by PCR using primers BamHI-fumI-for (5'-AGC TAT **GGA TCC ***ATG *GCG AAC GGA ACA AGG C-3') and PstI-Stop-fumI-rev (5'-CGC CTT **CTG CAG ***TCA *AGC ACC GGC GAG TTG-3'), digested with *Bam*HI and *Pst*I, and cloned into the same sites of vector pMAL-c2E. For cleavage of MBP (42.6 kDa) from the fusion protein (89.5 kDa) after affinity purification, the recognition site DDDDK for recombinant enterokinase, which is located upstream of the polylinker site in pMAL-c2E, was used.

### Expression of the aminotransferase gene

#### Expression from pET vectors

Cultures were grown in Luria-Bertani (LB) medium (5 g/l yeast extract, 10 g/l tryptone, 10 g/l NaCl) supplemented with 50 μg/ml ampicillin to select for plasmids derived from pET-3a, or 50 μg/ml kanamycin for plasmids derived from pET-30a(+) following the guidelines provided by Novagen. Initially, the *fumI *gene was expressed in *E. coli *BL21(DE3) from plasmid pET-30a-AT144 by using a 2% inoculum of an overnight culture, incubation at 37°C and 200 rpm until OD_600 _reached 1.0, addition of IPTG to a final concentration of 1 mM, and continued incubation for 3 h. Cells were harvested by centrifugation. For plasmid pET-3a-HISAT, several *E. coli *strains were compared with protein expression performed as above, except that induction was done with 0 μM, 10 μM, 50 μM, 100 μM, 200 μM, 400 μM or 1000 μM IPTG for *E. coli *Tuner, and 400 μM IPTG or no IPTG for all other strains, and temperature was shifted to 30°C after induction.

#### Expression as fusion protein with maltose-binding protein

Strains harboring plasmid pMAL-AT144 were grown overnight at 37°C and 250 rpm in LB-medium with 0.2% glucose and 50 μg/ml ampicillin, diluted 50-fold in the same medium, and incubated under the same conditions until OD_600 _reached 0.4 - 0.6. IPTG was added to a final concentration of 0.3 mM, and incubation was continued for 2 h at 37°C, or for 20 h at 30°C or room temperature (23.5°C).

#### Expression in E. coli ArcticExpress(DE3)

Cultivation conditions followed the guidelines provided by Stratagene. Strains harboring pET-3a or pET-30a(+) derived plasmids were grown overnight in LB medium with 20 μg/ml gentamycin and the appropriate antibiotic for the pET vector at 37°C and 250 rpm. The cultures were diluted 50-fold in LB medium without antibiotics and incubated for 3 h at 30°C and 250 rpm. The cultures were then transferred to 11°C (Ecotron incubator, Infors HT, Bottmingen, CH), and after 10 min of temperature equilibration, IPTG was added to a final concentration of 1 mM for pET-30a(+) or 0.4 mM for pET-3a derived plasmids. The induced cultures were incubated at 11°C and 250 rpm for 24 h. For subsequent chromatographic purification, *fumI *was expressed from plasmid pET-30a-AT144HIS as above, except that a modified M9ZB medium [66] was used, containing the M9 salts, 10 g/l tryptone instead of NZ-amine, 5 g/l yeast extract, 1 mM MgSO_4 _and 0.4% glucose.

### Purification of the recombinant aminotransferase

#### Purification of the MBP-aminotransferase fusion protein (MBP-FumI)

Pellets of 250-ml cultures were suspended in 10 ml of binding buffer (20 mM Tris-HCl, 200 mM NaCl, 1 mM EDTA, pH 7.4) and lysed by passing twice through a French Press (Thermo Electron Corporation, Waltham, MA, USA) at 20,000 psi cell pressure. The lysate was clarified by centrifugation at 9,000 × g for 30 min and 4°C, filtered through a 0.45 μm filter (Millipore, Bedford, MA, USA), and diluted 5-fold with binding buffer. Using an ÄKTAprime system (GE Healthcare Life Sciences, Uppsala, Sweden), 24 ml of that protein solution were loaded at a flow rate of 0.5 ml/min onto two serially connected 1-ml MBP Trap HP columns (GE Healthcare) which were pre-equilibrated with 15 ml binding buffer. After washing with 30 ml binding buffer, the fusion protein MBP-FumI was eluted with 10 ml of elution buffer (20 mM Tris-HCl, 200 mM NaCl, 50 mM maltose, pH 7.4). Fractions of 0.5 ml were collected, analyzed by SDS-PAGE using a 10% polyacrylamide gel, and those containing MBP-FumI were pooled and dialyzed against recombinant enterokinase (rEK) cleavage buffer (20 mM Tris-HCl, 20 mM CaCl_2_, 50 mM NaCl, pH 7.4) for 20 h at 4°C. To cleave the MBP tag, dialyzed MBP-FumI (120 μg/ml) was incubated with rEK at room temperature (rEK:MBP-FumI ratio (unit:μg) = 1:40). After 6 h of incubation, an equal amount of rEK was added again, and incubation was continued for 18 h at room temperature. Samples for SDS-PAGE analysis were taken before addition of rEK, after 6 h and after 24 h. For separation into soluble and insoluble fractions, samples were centrifuged at 20,817 × g for 20 min at 4°C, and the pellet was resuspended in the same volume of rEK cleavage buffer.

#### Purification of the 6xHis-tagged aminotransferase by immobilized nickel affinity chromatography

For the development of a purification protocol, the cell pellets from 100-ml expression cultures of *E. coli *ArcticExpress(DE3), producing aminotransferase from pET-30a-AT144HIS with a C-terminal 6xHis tag, were resuspended in 40 ml of binding buffer (20 mM sodium phosphate, 0.5 M NaCl, pH 7.4) containing 20 mM imidazole, and lysed by passing through a French Press twice at 20,000 psi cell pressure. The lysate was centrifuged at 9,000 × g for 30 min at 4°C. The filtered (0.45 μM) supernatant was divided into three aliquots and imidazole was added to the aliquots to a final concentration of 20 mM (I), 60 mM (II) and 100 mM (III), respectively. A 1-ml HisTrap HP column (GE Healthcare), pre-equilibrated with 5 ml binding buffer, was loaded with 6 ml of an aliquot at a flow rate of 1 ml/min using the ÄKTAprime system. The column was washed with 10 ml (I), 12 ml (II) or 14 ml (III) of binding buffer containing 20 (I), 60 (II) or 100 mM (III) imidazole. Proteins were eluted by increasing the imidazole concentration to 500 mM in a linear gradient over 25 ml, and fractions of 1 ml were collected.

Purification at preparative scale was done by resuspending the cells from a 1-l culture (wet weight of cell pellet: 10 g) in 10 ml of binding buffer with 60 mM imidazole, and performing lysis, centrifugation and filtration as above. The clear lysate was doubled in volume with binding buffer containing 60 mM imidazole, and loaded at a flow rate of 1 ml/min onto a 1-ml HisTrap HP column which was pre-equilibrated with 10 ml binding buffer. After washing the column with 15 ml of binding buffer, the recombinant protein was eluted by using a linear gradient of 60 to 500 mM imidazole over 25 ml elution buffer. Fractions of 2 ml were collected, and those containing the protein were pooled, diluted with imidazole-free binding buffer to a final concentration of 60 mM imidazole, and applied again to the re-equilibrated column. Purification was performed as before, except that the gradient elution was done with 50 ml buffer. Fractions were analyzed by SDS-PAGE, those containing aminotransferase in electrophoretically homogeneous purity were pooled, and the protein concentration was determined by the Bradford assay.

### Protein analysis

For analysis of aminotransferase production and solubility, 1.5-ml samples of expression culture were centrifuged. The biomass was resuspended in 200 μl of resuspension buffer (10 mM Tris-HCl, 25 mM EDTA and 0.25% β-mercaptoethanol, pH 8.2), 50 μl of freshly prepared lysozyme (100 mg/ml) were added, and the mixture was incubated for 5 min at 37°C. After addition of 750 μl Triton X-100, the mixture was incubated for another 5 min at 37°C. To distinguish between soluble and aggregated protein, a 100-μl sample of the lysate was centrifuged at 20,817 × g for 20 min at 4°C. The complete lysate, supernatant, and pellet after resuspension in the same volume of 10 mM Tris-HCl buffer (pH 8.2), were analyzed by SDS-PAGE as total, soluble and insoluble protein fractions, respectively. The protein samples were mixed 1:1 with 2× sample buffer (0.125 M Tris-HCl, 4% SDS, 20% glycerol, 0.02% bromophenol blue, 2% β-mercaptoethanol, pH 6.8) and heated 5 min at 95°C. SDS-PAGE gel electrophoresis [67] was done according to standard procedures [65] with a Hoefer SE 260 mini-vertical gel electrophoresis unit (San Francisco, CA, USA). Staining of SDS-PAGE gels was done either with Coomassie Brilliant Blue R-250 or silver nitrate according to standard procedures [65], or with GelCode Blue Stain Reagent (Thermo Scientific, Rockford, IL, USA) according to the manufacturer's instructions. Molecular weight marker BenchMark™ Protein Ladder was provided by Invitrogen (Carlsbad, USA).

Western blotting [68] was carried out using the WesternPro Alkaline Phosphatase Chromogenic Detection Kit (Peqlab, Erlangen, Germany) with the PerfectBlue "Semi-Dry"-Electro Blotter (Peqlab) according to the manufacturer's instructions. Anti-His_6 _mouse monoclonal antibody was obtained from Roche (Mannheim, Germany) and was used at a final concentration of 0.4 μg/ml.

Protein concentrations were determined by the method of Bradford [69] using the reagent provided by Sigma. Bovine serum albumin (BSA) was used as standard.

### Enzyme activity assays

Biomass from *E. coli *expression cultures was harvested by centrifugation at 4,500 × g for 10 min at 4°C, resuspended in the same volume of 20 mM Tris-HCl, 50 mM NaCl, 2 mM CaCl_2 _pH 7.4, and homogenized by passing through a French pressure cell at 20,000 psi. Enzyme activity was determined in the same buffer, after doubling the volume, and using final concentrations of 5 μM HFB_1_, 20 μM pyridoxal phosphate (PLP) and 3 mM pyruvate.

Enzyme assays with purified MBP-FumI fusion protein and purified His-tagged FumI were carried out in 20 mM Tris-HCl buffer pH 8.0, containing 0.067% BSA, 15 μM HFB_1_, 20 μM PLP and 5 mM pyruvate. The final concentration of aminotransferase was 20 μg/ml. All reaction mixtures were incubated in a water bath at 25°C, and samples from the reaction were inactivated at 99°C for 10 min.

### Liquid chromatography-mass spectrometry (LC-MS)

In order to analyze samples from enzyme activity assays, LC-MS measurements were performed. Samples were centrifuged and 40-μl aliquots were dried at 50°C under a stream of nitrogen, dissolved in 200 μl of solvent containing ^13^C-FB_1 _as internal standard, and analyzed by injecting 50 μl onto a Zorbax Eclipse XDB-C8 column in an Agilent 1100 LC system coupled to an Agilent 1100 G1946 D mass spectrometer operating in the selected ion monitoring (SIM) mode with positive electrospray ionisation as previously described [1]. For integration of 2-keto-HFB_1 _into the method, a sample of HFB_1_, which was reacted to completion with purified His-tagged aminotransferase, was measured by HPLC-MS in scan mode. A novel peak with m/z 427 [M+Na]^+ ^as most intense ion and m/z 387 [M+H-H_2_O]^+ ^as second most intense ion appeared at 11.5 min. In the next step, fragmentor voltages were optimized by flow injection and m/z 427 and 387 were included into the original SIM method. Ions and fragmentor voltages were as follows: HFB_1 _quantifier: m/z 406.4, 120 V; HFB_1 _qualifier: m/z 334.4, 220 V; ^13^C-FB_1 _quantifier: 756.5, 150 V; 2-keto-HFB_1 _quantifier: m/z 427, 130 V; 2-keto-HFB_1 _qualifier: m/z 387, 90 V. For calibration, standard solutions of FB_1_, HFB_1 _and 2-keto-HFB_1 _in the range of 0.05 to 2 μM containing 200 μg/l ^13^C-FB_1 _as internal standard were used.

## Competing interests

A long term goal of the present work is to make the aminotransferase FumI commercially available through BIOMIN GmbH for enzymatic detoxification of fumonisins. However, while the information contained in the present manuscript is of interest for those using microbial cell factories for enzyme production, it is irrelevant for a commercial application of the enzyme. Therefore, the authors declare no competing interests.

## Authors' contributions

DoH and WDM designed the experiments and wrote the manuscript. DoH performed the experiments. SH contributed some of the plasmids and, together with RG, participated in the design of the research with a special focus on recombinant gene expression without IPTG. HES developed the method for analytical quantification of 2-keto-HFB_1_. GS provided helpful discussion and expertise on mycotoxin biotransformation. DiH participated in the design of the research with a special focus on the maltose-binding protein fusion protein. All authors participated in editing the manuscript and all have read and approved the final version.
